# Integrated bioinformatics and experimental validation identify ATF3 as a key gene in secondary brain damage after intracerebral hemorrhage

**DOI:** 10.1371/journal.pone.0328530

**Published:** 2025-07-18

**Authors:** Tao Cui, Jinbang Huang, Chaoyong Zhang, Bin Wang

**Affiliations:** 1 Department of Neurosurgery, The First Affiliated Hospital of Anhui Medical University, Hefei, China; 2 Department of Neurosurgery, The Taihe County People’s Hospital, Taihe, China; Rutgers: Rutgers The State University of New Jersey, UNITED STATES OF AMERICA

## Abstract

**Background:**

Secondary brain injury following intracerebral hemorrhage (ICH) is a critical clinical challenge, yet the molecular mechanisms driving neuronal damage remain poorly understood. This study investigates the role of the transcription factor ATF3 and its downstream effector, VASP, in mediating neuronal injury via the platelet activation pathways.

**Methods:**

Differential gene expression analysis of the GSE24265 dataset was conducted using the limma package in R to identify key regulators in hemorrhagic conditions. Protein–protein interaction network analysis and bioinformatic predictions were employed to pinpoint central regulatory nodes and downstream targets. Validation experiments utilized the HT22 mouse hippocampal neuronal cell line, combining ATF3 overexpression/knockdown, dual luciferase reporter assays, and functional assessments (propidium iodide/Calcein-AM staining, flow cytometry). Enrichment analysis linked identified targets to biological pathways.

**Results:**

ATF3 was significantly upregulated in hemorrhagic conditions and identified as a central regulatory node. Bioinformatic and experimental validation confirmed VASP as a direct downstream target of ATF3. Enrichment analysis revealed VASP’s predominant association with platelet activation pathways. Functional assays demonstrated that ATF3 overexpression exacerbated heme-induced cytotoxicity in HT22 cells, implicating hyperactive platelet activation in secondary neuronal damage.

**Conclusions:**

This study identifies a novel ATF3–VASP signaling axis as a key driver of secondary neuronal injury post-ICH. Our findings advance the mechanistic understanding of post-hemorrhagic brain damage and suggest that therapeutic targeting of the ATF3–VASP pathway may mitigate secondary injury, offering a potential strategy to improve clinical outcomes.

## Introduction

Intracerebral hemorrhage (ICH), also known as hemorrhagic stroke, ranks among the most common causes of death and long-term disability worldwide [1]. Epidemiologically, ICH accounts for approximately 10–15% of all strokes but results in more than 40% of stroke-related deaths within 30 days of onset [2]. The Global Burden of Disease (GBD) analyses from 1990 to 2019 highlight that while ischemic stroke incidence has declined in many high-income countries, the burden of ICH remains stagnant or has worsened in low- and middle-income regions, notably in Asia and sub-Saharan Africa [3–5]. In China, for example, ICH contributes significantly to the national stroke burden due to a combination of hypertension prevalence, air pollution exposure, and limited access to preventive care [5]. The 2025 World Stroke Organization fact sheet [6] emphasizes the continued global challenge posed by ICH, calling for urgent strategies to enhance early diagnosis, blood pressure management, and equitable healthcare access. Despite advances in neuroimaging and surgical intervention, research into effective pharmacologic treatments for ICH remains limited, with few breakthroughs in clinical trials, highlighting the need for intensified global research investment [7].

Despite considerable progress in acute stroke management, the global burden of stroke remains substantial, underscoring the limitations of current therapeutic strategies [3]. In intracerebral hemorrhage (ICH), secondary brain injury (SBI) has emerged as the predominant determinant of long-term neurological disability and death [8]. The multifaceted and incompletely understood biological pathways underlying SBI—including neuroinflammation, oxidative stress, and blood-brain barrier disruption—have posed significant barriers to the development of effective neuroprotective treatments [9]. Therefore, advancing our understanding of the molecular and cellular mechanisms driving SBI is crucial to inform the design of targeted, mechanism-based interventions for ICH patients.

SBI, which ensues after the initial hemorrhagic insult, comprises a series of complex and multifactorial pathological cascades that extend beyond the primary damage. These events include robust inflammatory responses mediated by the activation of the coagulation cascade [10], neurotoxicity arising from the breakdown products of red blood cells [11], and oxidative stress driven by the liberation of free iron following hemoglobin degradation [12]. Additional mechanisms such as ferroptosis—a form of regulated cell death dependent on iron and lipid peroxidation [13]—and neuronal apoptosis induced by endoplasmic reticulum (ER) stress [14] further exacerbate tissue injury. Notably, the extravasation and lysis of erythrocytes result in the deposition of hemoglobin, heme, and iron ions in the brain parenchyma, which collectively serve as potent triggers of neuroinflammation and secondary neuronal damage [15–18]. To elucidate the molecular basis of these pathologies, transcriptomic analyses of human brain tissue from cases of spontaneous ICH identified Activating Transcription Factor 3 (ATF3) as a candidate gene potentially involved in ICH pathogenesis. This discovery enhances our understanding of post-ICH molecular alterations and provides a basis for exploring ATF3 as a novel therapeutic target.

## Materials and methods

### Microarray gene expression

The Gene Expression Omnibus (GEO) database (http://www.ncbi.nlm.nih.gov/geo/) provides gene expression profiles under series number GSE24265, generated using the GPL570 platform. Therefore, dataset GSE24265, which includes gene expression data from four perihematomal tissue samples, four contralateral white matter samples, and contralateral gray matter samples from patients with ICH, was retrieved from the GEO database in the present study. Based on the platform’s annotation information, probe IDs were mapped to their corresponding gene symbols, and duplicate gene names were resolved using the mean value method.

RNA-seq data on human brain tissue following intracerebral hemorrhage are from GSE24265, which was approved by the relevant ethics committee during the sampling procedure. In this study, we reuse the dataset submitted without prejudice to ethical considerations of the reuse of the dataset.

### Identification and selection of differentially expressed genes (DEGs)

The limma package in R/Bioconductor was utilized to identify differentially expressed genes (DEGs) based on the criteria P < 0.05 and |log₂ (fold change) | ≥ 1.5. Hierarchical clustering and data visualization were performed using the Heatmap package in R.

### Functional enrichment analysis

Functional enrichment analysis of DEGs was conducted using the clusterProfiler package in R. Based on the expression levels of key genes, ICH samples were categorized into high- and low-expression groups. Gene Set Enrichment Analysis (GSEA, version 4.2.3) was then performed to evaluate differences in biological states between these groups. The background gene set data were obtained from the Molecular Signatures Database (MSigDB) v7.5.1, specifically utilizing the Kyoto Encyclopedia of Genes and Genomes (KEGG) subset of C2 and the Gene Ontology (GO) subset of C5 for functional enrichment analysis. Pathways with a false discovery rate (FDR) < 25% and nominal P < 0.05 were considered significantly enriched [19].

### Establishment of PPI networks and identification of hub genes

PPI networks of DEGs were evaluated using STRING. Cytoscape V3.9.1 was used to visualize the PPI network of key DEGs. MCODE in Cytoscape was used to screen out the hub cluster.

### Transcription factor–DEG network construction

TF–DEG interaction data were obtained from several databases, including KnockTF, hTFtarget, JASPAR, ChIP_Atlas, GTRD, and ENCODE. Differentially expressed genes that exhibited reciprocal changes in transcription factor levels were selected, and the corresponding regulatory TFs were subsequently identified. An integrated TF–DEG network was then constructed and visualized using Cytoscape.

### Establishment of ICH mice

To establish a mouse model of ICH, adult C57BL/6 mice (male, 8–10 weeks old) were housed under standardized conditions (12-hour light/dark cycle, 22°C, 50% humidity) with ad libitum access to food and water, supplemented with environmental enrichment (nesting material, social housing). All procedures were approved by the Anhui Medical University Institutional Animal Care and Use Committee (IACUC一AHMU 2024-Y-1099) and conducted in accordance with ARRIVE guidelines. Mice were anesthetized with 3% isoflurane (induction) followed by 1.5% isoflurane maintenance in 70% N₂O/30% O₂, secured in a stereotactic frame, and injected with 25 µL autologous blood into the striatum (coordinates: AP + 0.2 mm, ML ± 2.0 mm, DV −3.0 mm from bregma) using a microinjection pump (0.5 µL/min). The needle remained in place for 10 minutes post-injection to prevent backflow. Sham controls received equivalent saline injections. Postoperative care included ① subcutaneous meloxicam (5 mg/kg) administered preoperatively and every 24 hours for 72 hours for analgesia. ② Lactated Ringer’s solution (1 mL, subcutaneous) immediately post-surgery to maintain hydration. ③ Twice-daily monitoring for neurological deficits (asymmetry, lethargy, weight loss ≥20%, or inability to access food/water). Humane endpoints were defined as severe distress (e.g., labored breathing, prolonged immobility), uncontrolled seizures, or weight loss >20% over 48 hours. Animals meeting these criteria were euthanized immediately via isoflurane overdose (5% for 5 minutes) followed by transcardial perfusion. All surviving mice were euthanized under deep anesthesia (5% isoflurane) at 24, 48, or 72 hours for histological validation (H&E staining). Tissue collection was performed after confirmation of death via cervical dislocation and bilateral pneumothorax. Veterinary staff monitored animals daily, with no unexpected deaths or adverse events occurring during the study. The animal experiment was approved by the Animal Management and Use Committee of the First Affiliated Hospital of Anhui Medical University (NO. 2024133) and the management and use of mice are consistent with the relevant guidelines of US National Institutes of Health. The study is reported in accordance with ARRIVE guidelines.

### Neurofunctional and behavioral assessment

Sensorimotor performance was assessed using a 28-point neurological score, the corner test, and forelimb placement tasks, administered pre-surgery and at 48 hours post-injury [20,21]. All evaluations were performed by an observer unaware of the treatment groups.

### Neurological evaluation

Acute neurological impairments were measured with a 28-point scale covering aspects such as body symmetry, gait, climbing ability, circling behavior, forelimb symmetry, involuntary circling, and whisker reflex. Each criterion was scored from 0 to 4, with a total possible score of 28 indicating the most severe deficit [20].

### Forelimb placement test

This behavioral assessment involved gently holding the animals by the torso, allowing the forelimbs to hang freely. To promote muscle relaxation, the animals were softly moved up and down prior to testing. Each forelimb was examined by brushing the vibrissae on the respective corner of a countertop. Healthy animals promptly positioned the corresponding forelimb onto the edge of the surface following vibrissae stimulation. Each animal underwent 10 trials per forelimb, and the percentage of successful placements was calculated based on the number of trials where the correct forelimb was placed after stimulation.

### Corner test

In this assay, a mouse was guided into a corridor leading to a 30° turn. To exit, it could choose to turn either left or right. The direction of each turn was recorded. The counts of right and left turn out of 10 attempts were documented. The laterality index (LI) and normalized LI were determined following Bouet et al. [22]. The LI for each subject was calculated using:


LI=(numberofrightturns–numberofleftturns)/totalturns.

The LI on the day before surgery (LIBS) and on subsequent post-surgical days was used to compute a normalized LI:


NormalizedLI=(LI+2)/(LIBS+2).

### Histopathologic analysis and lesion volume measurement

Following euthanasia, brains were perfused with 4% paraformaldehyde in 0.1 mol/L PBS (pH 7.4), rapidly extracted as intact tissue, and then fixed in 4% paraformaldehyde for 12 hours at 4°C. They were subsequently immersed in 30% sucrose for 2–3 days at 4°C. The brains were then embedded in Optimal Cutting Temperature (OCT) compound (Sakura Finetek, No. 4583), frozen on dry ice, and stored at –80°C. Coronal sections, 20 micrometers thick, were prepared using a cryostat and stained with Nissl stain. To calculate the striatal lesion volume, serial coronal sections spaced 50 μm apart were digitized to encompass the entire hematoma. A blind observer delineated the hematoma area based on blood presence within the striatum. The total lesion volume (mm^3^) was obtained by summing the areas of the hematoma in each section and multiplying by the section thickness (0.5 mm).

### Determination of brain water content

Three days after hemorrhage, mice were anesthetized with ketamine (80–100 mg/kg, intraperitoneally) and xylazine (5–10 mg/kg, intraperitoneally), then euthanized to measure brain water content (sham n = 6; vehicle n = 8; ATF3 overexpressed mice n = 8; ATF3 downregulated mice n = 11). Immediately, brains were removed as a whole, and five 2-mm coronal slices were prepared starting 2 mm anterior to the frontal pole. Each slice was split into hemispheres along the midline; the cortex was carefully dissected from underlying basal ganglia in each hemisphere, with the cerebellum retained as a control. The wet weight of each section was measured using an electronic balance (Model AG 104; Mettler-Toledo). The sections were then placed on preweighed cover slips and dried overnight in a vacuum oven for 24 hours to determine the dry weight. Brain water content (%) was calculated using: ((wet weight – dry weight)/ wet weight) × 100.

### Cell culture and treatment

HT-22, a mouse hippocampal neuronal cell line, was acquired from SIGMA MERCK (catalog number SCC129) and authenticated by short tandem repeat (STR) analysis conducted by Shanghai Biowing Applied Biotechnology Co. Ltd. Cells were maintained in DMEM/F12 medium supplemented with 100 U/mL penicillin, 100 μg/mL streptomycin, and 10% fetal bovine serum (FBS) at 37°C in a humidified incubator with 5% CO₂, with the medium refreshed every two days prior to experimentation.

For experimental treatments, cells were seeded at densities of 1 × 10⁴ cells per well in 96-well plates, 2 × 10⁴ cells per well in 24-well plates, 2 × 10⁵ cells per well in 6-well plates, and 5 × 10⁵ cells per 6-cm dish. One day post-seeding, HT-22 cells were exposed to heme (dissolved in DMEM at a concentration of 80 μM) for 24 hours. Unless otherwise specified, identical coating conditions were applied in all experiments.

### Propidium iodide (PI)/Calcein-AM staining

The percentage of dead cells was detected using a PI/Calcein-AM double staining kit (Shanghai, China). Calcein-AM (10 μM, C3099, Invitrogen) and PI (10 μM, P3566, Molecular Probes) were added to HT22 cells and incubated at 37°C for 20 min after the necessary treatment. A fluorescence microscope (Olympus IX73, Tokyo, Japan) was used to capture the images. Excitation filters of 490 nm and 545 nm were used to observe the living and dead cells, respectively.

### Western bolt

Total protein extracts from HT22 cells and mouse brain tissue were prepared as previously described [23,24]. Protein concentrations were quantified using the BCA Protein Assay Kit (Pierce). The extracted proteins were then separated via 10% sodium dodecyl sulfate-polyacrylamide gel electrophoresis (SDS-PAGE) and transferred onto Immobilon-P membranes (Millipore, Billerica, MA). Following transfer, the membranes were blocked with 5% skim milk at room temperature for 1 hour and subsequently incubated with primary antibodies at 4°C overnight. The next day, the membranes were incubated with horseradish peroxidase (HRP)-conjugated secondary antibodies for 1 hour at room temperature. Protein signals were detected using a chemiluminescence reagent kit (Millipore) and analyzed with Image-Pro Plus 6.0 software. GAPDH (Cell Signaling Technology, USA) was used as a loading control.

### Immunofluorescence

HT22 cells were plated in serum-free medium (SFM) at a density of 10⁴ cells/cm^2^ on sterile round glass coverslips in 12-well plates. Two days post-seeding, fresh steroid-free medium containing heme was added, as specified in the Results section. After 24 hours of incubation, cell monolayers were rinsed with Dulbecco’s phosphate-buffered saline (DPBS) and fixed with 4% paraformaldehyde (PFA) in DPBS for 15 minutes. Following fixation, PFA was replaced with DPBS, and cultures were stored at 4°C until immunostaining.

For immunostaining, cells were incubated with either a mouse monoclonal ATF3 antibody (Abcam, ab254268) or a rabbit polyclonal VASP antibody (Proteintech, 13472-1-AP, China) as primary antibodies. Primary antibody incubation was performed for 60 minutes at a 1:50 dilution in PBS–CAS. Subsequently, cells were incubated with CoraLite594-conjugated goat anti-mouse/rabbit IgG (H + L) (Proteintech, China) as the secondary antibody. After final washes in PBS, coverslips were mounted onto glass slides using a commercial antifading medium (Vectashield, Vector Laboratories). Negative controls were prepared by omitting the primary antibody.

### Flow cytometry

Flow cytometry was employed to quantify apoptosis following heme treatment, using an Annexin V-FITC kit on the FACS Santo II (BD, USA), as previously described [25]. HT22 cells were incubated with 80 μM heme for 16 hours.

### Statistical analysis

Data are presented as the mean ± standard deviation (SD). Statistical analyses were performed using GraphPad Prism v9.1.1. Comparisons between two groups were conducted using a two-tailed independent-sample Student’s T-test, while differences among multiple groups were assessed using one-way analysis of variance (ANOVA). Pearson’s correlation coefficient was applied to evaluate the relationship between two variables. A bilateral p < 0.05 was considered statistically significant.

## Results

### Data normalization

**Fig 1:**
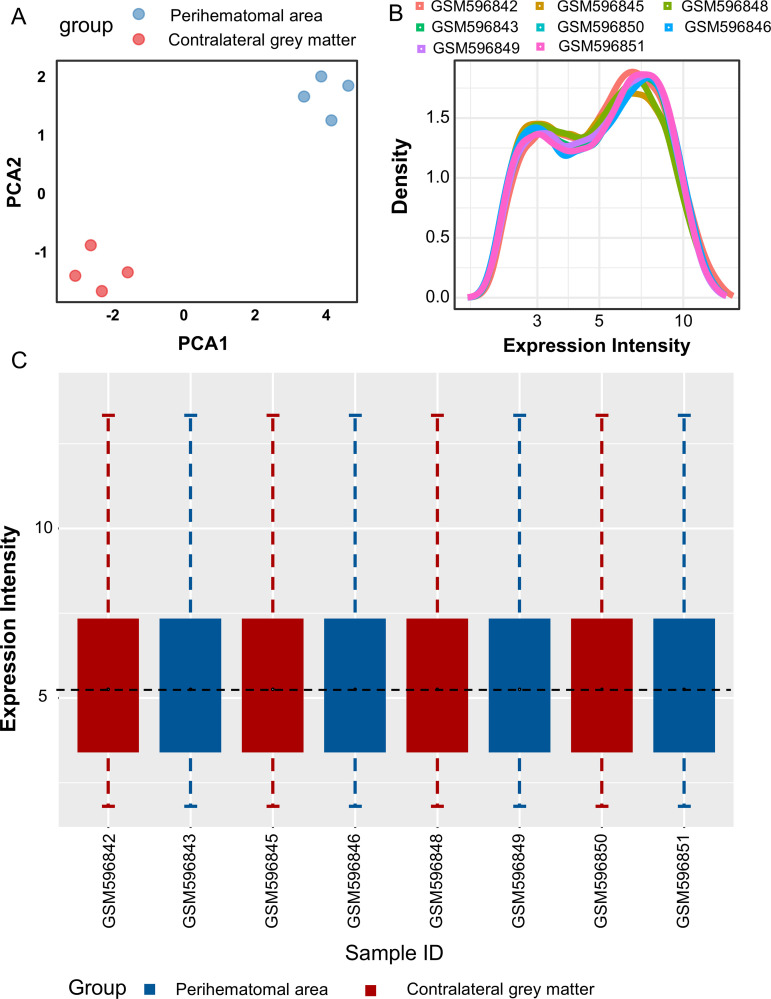
The distribution characteristics of expression levels in the samples after normalization. (A) Principal component analysis (PCA) of log2-transformed and mean-centered expression data, displaying the first two principal components (PC1: 58.3% variance; PC2: 22.1% variance). Samples are color-coded by tissue origin (blue: perihematomal area, red: contralateral grey matter), with 95% confidence ellipses calculated using multivariate t-distribution. (B) Gaussian kernel density estimation (bandwidth = 0.15) showing bimodal distribution of normalized expression values (Kolmogorov-Smirnov test against normal distribution: D = 0.21, p < 0.001), indicating tissue-specific expression subpopulations. (C) Tukey-style boxplot comparing expression intensity distributions across samples, with whiskers extending to 1.5 × interquartile range (IQR). Outliers (circles, > Q3 + 1.5IQR) were identified using Benjamini-Hochberg corrected thresholds (FDR < 0.05). Horizontal lines denote median values with significant inter-group differences.

The microarray dataset GSE24265 was retrieved from the GEO database, comprising 11 brain tissue samples (4 from the perihematomal area and 7 from contralateral grey matter and contralateral white matter). Data normalization and cross-comparability assessments were performed to mitigate technical and systematic biases. Principal component analysis (PCA) was conducted to assess biological variability among the samples. The resulting PCA plot demonstrated distinct clustering of PBC and HC samples, reflecting divergent gene expression profiles (Fig 1A). The density plot indicated that gene expression distributions across samples from different groups followed similar patterns, supporting their inclusion in subsequent analyses (Fig 1B). The box plot revealed a consistent range of gene expressions for each sample, with the median values represented by black lines closely aligned within the boxes (Fig 1C). Robust normalization of raw data ensured the reliability of the data for downstream analyses.

### Identification of DEGs

**Fig 2:**
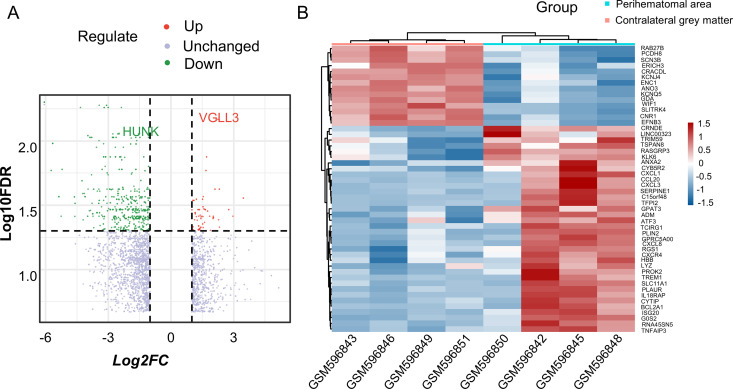
Volcano plot and heatmap of DEGs in the datasets. (A) Volcano plot of all differentially expressed genes (DEGs) in GSE24265, analyzed using the limma package in R. A threshold of adjusted p-value < 0.05 and |log₂ fold change (FC)| ≥ 1.5 was applied to identify significant DEGs. (B) Heatmap of the top 55 DEGs, comprising 41 upregulated and 14 downregulated genes. DEGs, differentially expressed genes.

Using the limma package in R (with |logFC| ≥ 1.5 and an adjusted p-value < 0.05), we identified 1208 differentially expressed genes (DEGs), comprising 263 upregulated and 945 downregulated genes (S1 Table). The volcano plot (Fig 2A) and the heatmap (Fig 2B) illustrate these expression differences, highlighting significant disparities between the two groups.

### Functional enrichment analysis of DEGs

**Fig 3:**
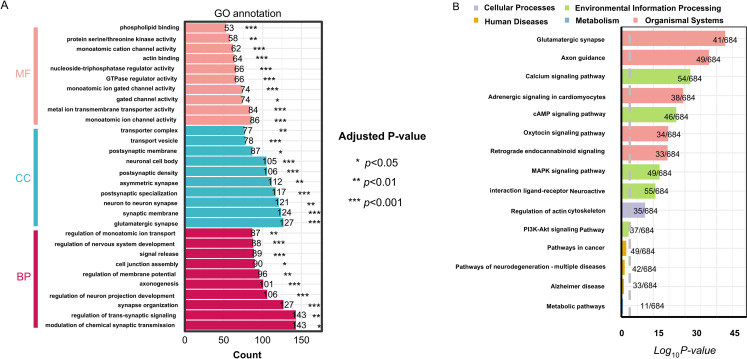
Functional enrichment analysis of differentially expressed genes (DEGs). **(A)** Gene Ontology (GO) analysis, including biological processes (BPs), molecular functions (MFs), and cellular components (CCs) of DEGs. **(B)** Kyoto Encyclopedia of Genes and Genomes (KEGG) pathway analysis of DEGs. GO, gene ontology; DEGs, differentially expressed genes; BP, biological process; MF, molecular function; CC, cellular component, KEGG, Kyoto Encyclopedia of Genes and Genomes.

To elucidate the biological functions of the identified DEGs, Gene Ontology (GO) enrichment analysis was conducted using clusterProfiler (Fig 3A, S2 Table). The DEGs were significantly enriched in biological processes (BPs) related to the regulation of monoatomic ion transmembrane transport, membrane potential modulation, trans-synaptic signaling, and chemical synaptic transmission. In terms of molecular functions (MFs), the DEGs were primarily associated with metal ion transmembrane transporter activity, GTPase regulator activity, phospholipid binding, monoatomic cation channel activity, and gated channel activity. Regarding cellular components (CCs), enrichment was observed in the transporter complex, postsynaptic membrane, and neuron-to-neuron synapse.

To further explore the functional relevance of these DEGs, Kyoto Encyclopedia of Genes and Genomes (KEGG) pathway enrichment analysis was performed (Fig 3B, S3 Table). The DEGs were predominantly enriched in neurotransmitter transmission signaling pathways, including the glutamatergic synapse, axon guidance, calcium signaling pathway, and cAMP signaling pathway. These findings suggest that the identified DEGs play a crucial role in dysfunction of neurotransmitter transmission, further supporting the hypothesis that neuronal cell dysfunction is a key factor in the pathogenesis of ICH.

### PPI network construction of DEGs

**Fig 4:**
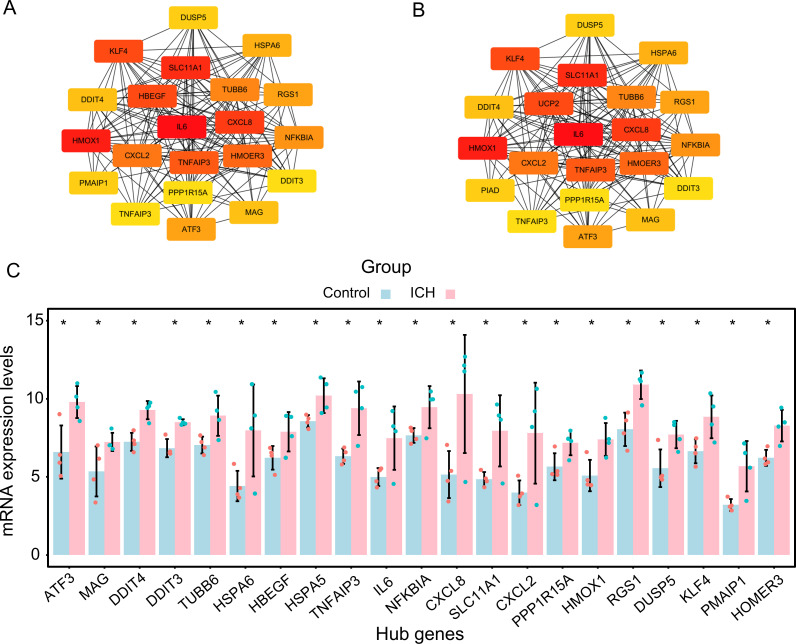
Protein–Protein Interaction (PPI) Network and the Most Significant Module of DEGs. **(A)** The most significant module was identified from the PPI network using cytoHubba in Cytoscape. **(B)** The most significant module was also identified using MCODE in Cytoscape. **(C)** Expression changes of 21 hub genes between the perihematomal area and contralateral grey matter samples (GSE24265). PPI, protein–protein interaction; DEGs, differentially expressed genes.

The STRING database has been used to build a PPI network that can illustrate the relationships between protein interactions encoded by DEGs. According to the results, there were 159 nodes and 470 edges in the PPI network (S1 Fig). Of note, most of the proteins encoded by DEGs were highly interlinked with others. Of them, the most significant module, which included 21 genes, was screened out from the PPI network based on module analysis using the MCODE and cytoHubba (Fig 4A and 4B). The DEGs in the module included ATF3, MAG, DDIT4, DDIT3, TUBB6, HSPA6, HBEGF, HSPA5, TNFAIP3, IL6, NFKBIA, CXCL8, SLC11A1, CXCL2, PPP1R15A, HMOX1, RGS1, DUSP5, KLF4, PMAIP1, and HOMER3. Moreover, changes in mRNA expression levels of 21 module genes differed significantly between perihematomal area and contralateral gray matter samples (Fig 4C). Of note, several DEGs, including DDIT4, TUBB6, HBEGF, HSPA5, TNFAIP3, IL6, CXCL8, CXCL2, HMOX1, RGS1, NFKBIA, and KLF4, have been widely studied in the pathogenesis of ICH [26–36]. However, ATF3, the focus of this study, has not yet been reported. The findings revealed that not only was ATF3 a notably upregulated DEG in ICH, but also one of the hub genes in the PPI network of the DEGs.

### Construction of the TF–hub genes network

**Fig 5:**
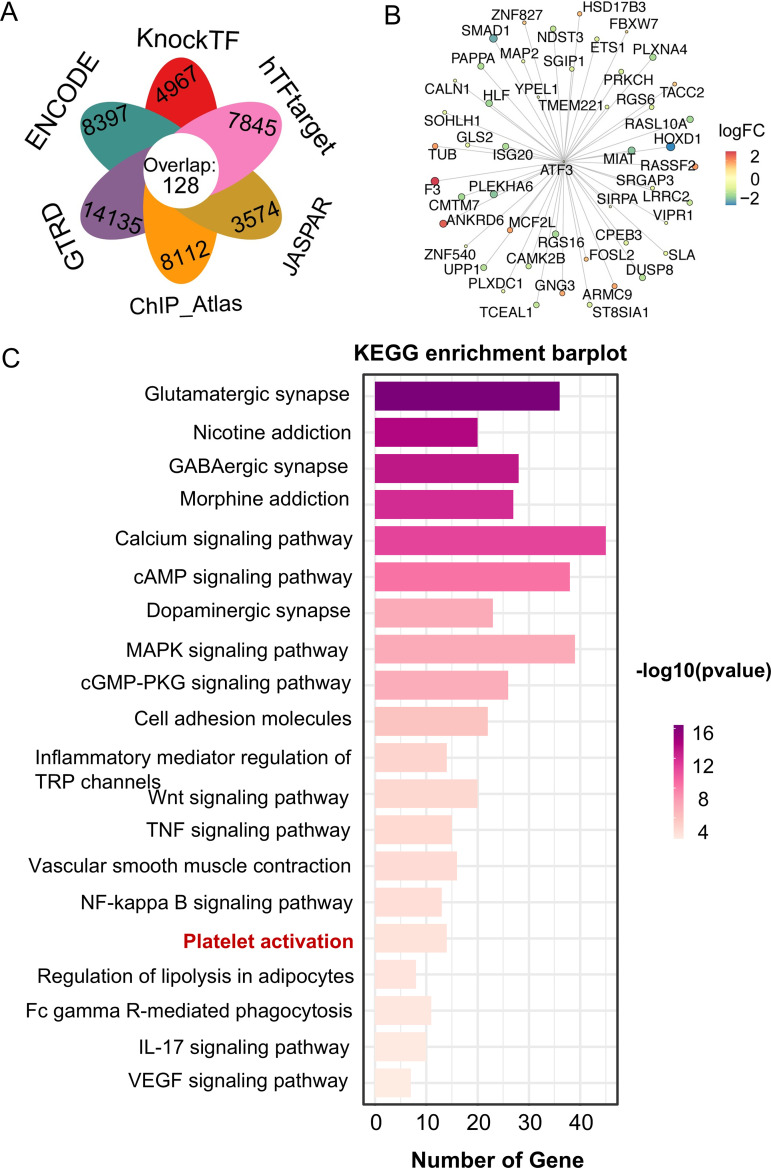
Enrichment analysis of ATF3 target genes and their related functions. **(A)** Venn plot for target gene prediction of ATF3 based on ENCODE, KnockTF, hTFtarget, JASPAR, ChIP_Atlas, and GTRD databases. **(B)** ATF3-DEGs networks. **(C)** Kegg pathways enrichment for ATF3 target genes and genes with closely related expression levels.

To investigate the functional roles of ATF3, the potential regulatory relationship between DEGs and ATF3 was explored. The potential regulatory relationships between DEGs and ATF3 were screened based on TF-binding site data and genetic coordinate position information provided on ENCODE. We found that the ATF3–DEGs interaction network included 128 target DEGs (Fig 5A and 5B). Finally, to explore the biological functions of ATF3 and its target genes, we further performed GO and KEGG pathway enrichment analysis. Our results indicated that genes closely related to ATF3 were enriched in the glutamatergic synapse, GABAergic synapse, calcium signaling pathway, TNF signaling pathway, platelet activation, and so on (S2 Fig). The results of enrichment analysis of KEGG pathways indicated that the inflammation pathways (e.g., NF-kappa B, TNF-α, and IL-17-signaling pathways), MAPK signaling pathways, cell adhesion molecules, and platelet activation were significantly enriched in perihematomal area tissues (Fig 5C). Of note, the involvement of platelet activation in secondary brain injury following intracerebral hemorrhage (ICH) has been investigated; however, the regulatory mechanism of ATF3 in ICH remains unexamined.

### Validating the expression of ATF3 in vitro and vivo

**Fig 6:**
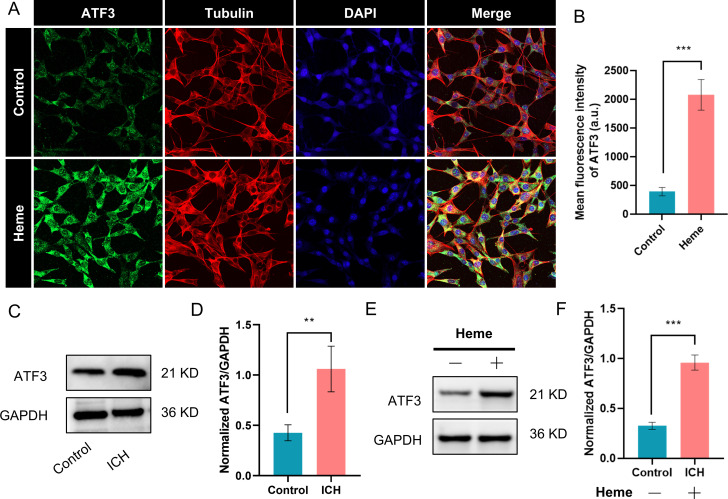
Heme-induced ATF3 activation in cellular and murine models of intracerebral hemorrhage (ICH). **(A)** Immunofluorescence of HT-22 cells (80 μM heme, 24 hr) showing nuclear ATF3 accumulation (red; rabbit anti-ATF3, Cell Signaling 33593, 1:200) vs. vehicle control (0.1% DMSO). Nuclei counterstained with Hoechst 33342 (blue). Scale bars: 20 μm. **(B)** Quantification of ATF3 fluorescence intensity (n = 6 fields/group; ImageJ v1.53; unpaired t-test, **p = 0.0003). Data normalized to control (dashed line). **(C)** Western blot of perihematomal brain lysates (72 hr post-ICH). Blots: Rabbit anti-ATF3 (1:1,000), GAPDH loading control (mouse monoclonal, Sigma A2228, 1:5,000). **(D)** Densitometric analysis (n = 5 mice/group; one-way ANOVA, *p = 0.0048 vs. sham). Normalized to GAPDH. **(E)** In vitro validation: ATF3 expression in heme-treated HT-22 cells (80 μM, 24 hr). **(F)** Quantitation of ATF3 protein levels (n = 4 biological replicates; ordinary one-way ANOVA, *p = 0.0071 vs. control).

The results presented above demonstrate a significant increase in the expression of ATF3 mRNA in the brain tissue surrounding the hematoma. To further validate these expression changes, we conducted a series of experiments. The results of immunofluorescence staining revealed that the treatment of HT22 cells with 80 μM heme resulted in a substantial increase in ATF3 expression (Fig 6A and 6B). Western blot also confirmed that ATF3 expression was significantly elevated in both the perihematomal brain tissues from the ICH mouse model (Fig 6C and 6D) and in HT22 cells treated with 80 μM heme (p < 0.05) (Fig 6E and 6F). These findings provide additional support for the conclusion that ICH leads to an upregulation of ATF3 expression in the local brain tissue of the hematoma following ICH.

### Upregulated ATF3 expression promoted VASP transcription

**Fig 7:**
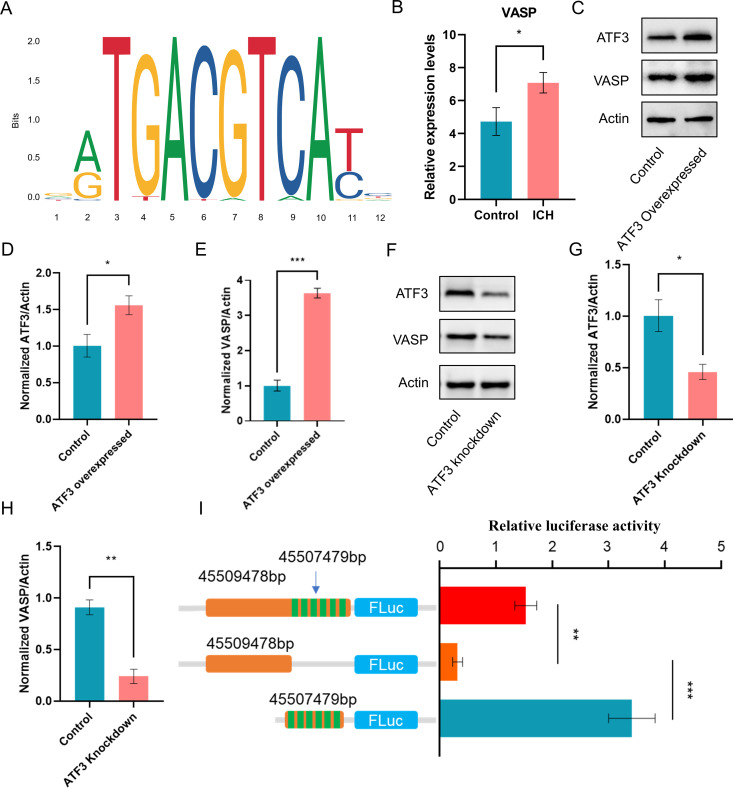
Mechanistic validation of VASP as a direct transcriptional target of ATF3. **(A)** Evolutionary-conserved ATF3 binding motif identified by JASPAR (Matrix ID MA0606.1) within the VASP promoter (−1.5 kb upstream of TSS). Position frequency matrix conservation score >85% across mammals. **(B)** Reanalysis of GSE24265 microarray data showing VASP upregulation (linear models for microarray data [LIMMA], adj.p = 0.017). **(C)** ATF3 gain-of-function model: Western blot of HT-22 cells 48 h post-lentiviral ATF3 overexpression (pLVX-EF1α vector). Blots: Rabbit anti-ATF3 (CST 33593, 1:1,000), mouse anti-VASP (BD 611175, 1:500), β-actin control (Sigma A2228, 1:5,000). (D/E) Densitometric quantification of ATF3 (D) and VASP (E) protein levels (n = 3 biological replicates; unpaired t-test, **p = 0.0007 vs. EV control). **(F)** ATF3 loss-of-function: shRNA-mediated knockdown in HT-22 cells. Blots: 72 h post-transduction; scramble shRNA control (Addgene #1864). (G/H) The quantification of ATF3 (G) and VASP (H) suppression (n = 3; two-way ANOVA, **p < 0.0001 vs. scramble). **(I)** Dual-luciferase reporter assay of VASP promoter activity. Left: Wild-type vs. ΔATF3-binding mutant constructs. Right: Firefly/Renilla ratio (n = 6; **p = 0.0004, ordinary one-way ANOVA).

Based on predictions from online databases, VASP was identified as one of the most promising downstream target genes of ATF3. As illustrated in Fig 7A, we characterized the binding sequence of ATF3 on the VASP gene. Moreover, the VASP mRNA expression level was markedly increased in perihematomal brain tissue (Fig 7A and 7B), suggesting a positive regulatory relationship between ATF3 and VASP. To validate this association, we assessed VASP expressions in cellular models following ATF3 overexpression and knockdown. Results showed that ATF3 overexpression resulted in increased VASP expression, while ATF3 knockdown resulted in decreased VASP expression (Fig 7C–7H). Finally, to confirm that ATF3 directly regulates VASP transcription, we constructed a dual luciferase reporter assay, which further corroborated the direct regulatory interaction between ATF3 and VASP (Fig 7I). Collectively, these findings indicate that VASP functions as a downstream target gene of ATF3.

### Increased expression of ATF3 leads to a significant increase in HT-22 cell death rate

**Fig 8:**
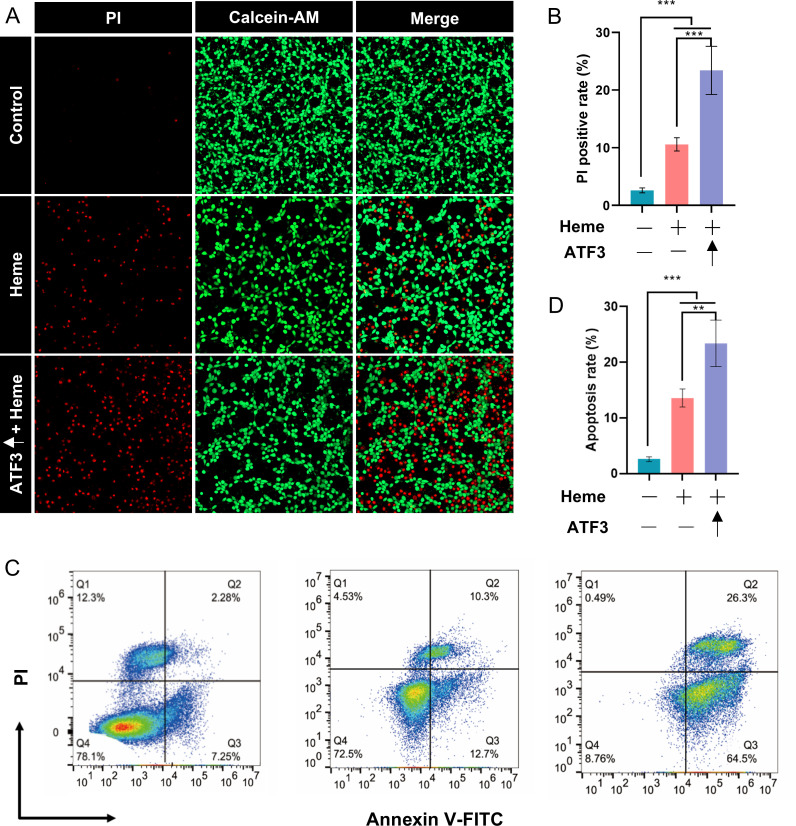
ATF3 overexpression exacerbates heme-induced cytotoxicity in HT-22 hippocampal neurons. **(A)** Live/dead cell imaging after 24 h heme exposure (80 μM): Calcein-AM (green, live cells; 2 μM, 30 min) vs. propidium iodide (PI, red, dead cells; 1 μg/mL). Scale bars: 50 μm (20 × objective). Inset: 63 × magnification of boxed region. **(B)** Quantification of PI⁺ cells (n = 8 fields/group; ImageJ particle analysis). Data normalized to vector control (dashed line). ATF3-overexpressing cells showed a 2.3-fold increase in mortality (unpaired t-test, **p = 0.0007 vs. empty vector + heme). **(C)** Annexin V-FITC/PI flow cytometry: Representative density plots of early (Annexin V ⁺ /PI⁻) and late (Annexin V ⁺ /PI⁺) apoptosis. Cells treated 24 h with 80 μM heme (Sigma H9039, ≥ 90% purity). **(D)** Apoptotic index quantification (n = 5 biological replicates; BD FACSAria III). ATF3-OE increased total apoptosis to 26.3% vs. 2.28% in controls (two-way ANOVA, **p < 0.0001).

Finally, to investigate whether increased ATF3 expression following ICH contributes to local neuronal damage within the hematoma, we conducted a series of experiments. The PI/Calcein-AM staining revealed that ATF3 upregulation is associated with a significant increase in cell death in HT22 cells (Fig 8A and 8B), indicating that upregulation of ATF3 expression significantly increases the sensitivity of H-T22 to heme toxicity. Moreover, flow cytometry analysis further demonstrated that overexpression of ATF3 markedly elevates the apoptosis rate in these cells (Fig 8C and 8D). Collectively, these results suggest that heightened ATF3 expression in the hematoma-adjacent tissue is a key factor driving local neuronal cell damage following ICH.

### ATF3 overexpression leads to significant neurological deficits around the hematoma

**Fig 9:**
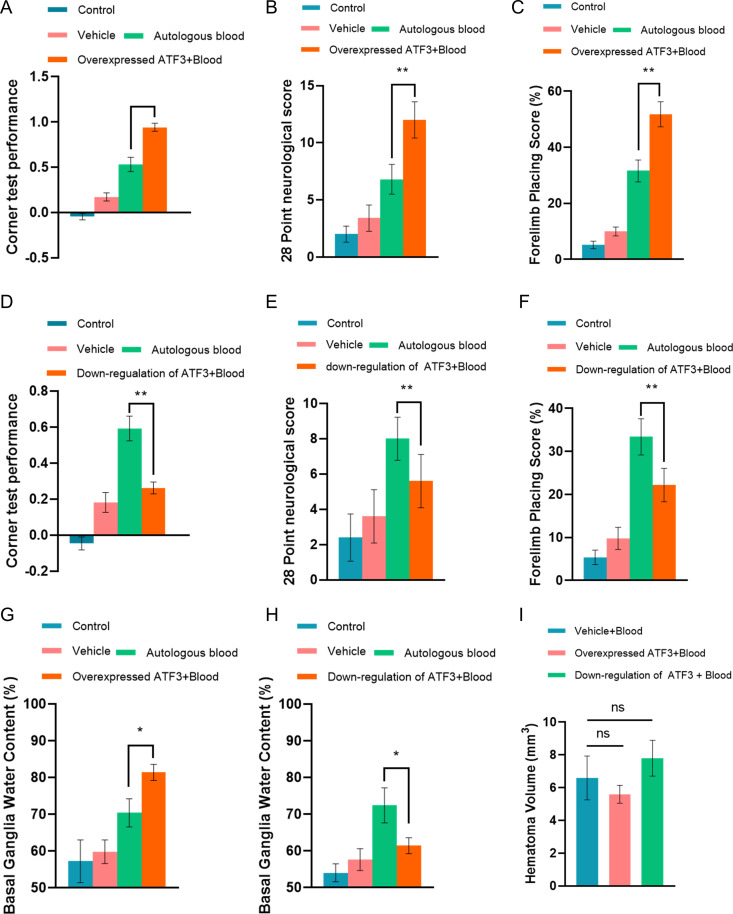
Effects of Alteration in ATF3 expression on neurological function and local brain edema in mice with ICH. (A, B and C). Compared with the only cerebral hemorrhage group, the Corner test score, 28 points neurological score and forelimb placing score of the cerebral hemorrhage model mice increased significantly, indicating that overexpression of the ATF3 gene led to aggravated neurological damage in the cerebral hemorrhage model mice; (D, E, and F). Compared with the only cerebral hemorrhage group, the Corner test score, 28 points neurological score and forelimb placing score in the ATF3 gene down-regulation group were significantly decreased, indicating that ATF3 gene down-regulation effectively improved the neurological function damage in cerebral hemorrhage model mice. **(G and H)**. Compared with the ICH model group, the water content in the basal ganglia of mice with ATF3 overexpression was significantly increased, while that in the ATF3 downregulation group was significantly decreased. **(I)**. Compared with the cerebral hemorrhage model group, there was no significant difference in the average hematoma volume between the ATF3 overexpression mice and the ATF3 downregulation group.

At 48 h after ICH there were marked functional deficits as assessed by the 28-point scoring system, corner turn test, and forelimb placing test (Fig 9A–9F). All tests showed a significant function deficit in the ATF3 overexpression group and a gradual recovery of function in mice with downregulated expression of ATF3 (n = 24). Additionally, compared with the control group, animals with downregulated expression of ATF3 also showed a significant reduction of basal ganglia water content, while a significant increase was observed in the ATF3 overexpression group (Fig 9G and 9H). Of note, forty-eight hours after ICH, there was no significant difference in the average hematoma volume between them (Fig 9I). This may be related to the injection of the same dose of autologous blood during the preparation of our cerebral hemorrhage model mice.

## Discussion

In this study, we analyzed the GSE24265 dataset to identify differentially expressed genes (DEGs) following cerebral hemorrhage, with a particular focus on ATF3, which exhibited a marked increase in expression. Our robust data processing using the limma package (|logFC| ≥ 1.5, adjusted p-value < 0.05) confirmed the significant upregulation of ATF3, consistent with its well‐established role as an immediate early gene in response to cellular stress and injury [37–41]. These findings align with earlier reports on the early transcriptional responses that occur after brain injury [42,43], underscoring the potential of ATF3 as a key molecular marker in the pathophysiology of secondary brain damage.

Network analysis via a protein–protein interaction (PPI) network construction revealed that ATF3 functions as a core regulatory node, orchestrating the expression of numerous DEGs. Bioinformatic predictions and subsequent experimental validations confirmed that ATF3 directly upregulates the downstream target VASP—a gene primarily associated with platelet activation [44–46]. Dual luciferase reporter assays further substantiated the direct transcriptional regulation of VASP by ATF3, and this regulatory relationship is supported by analyses of transcription factor databases [47–52]. Further, our enrichment analysis demonstrated that VASP is significantly linked to platelet activation—a process implicated in exacerbating secondary neuronal injury [53–56]. Experimental manipulation through gene overexpression and knockdown validated that ATF3 upregulates VASP expression, while dual luciferase assays confirmed the direct regulatory interaction. More importantly, functional validation through PI/Calcein-AM staining and flow cytometry demonstrated that elevated ATF3 expression significantly increases the susceptibility of HT22 neuronal cells to heme-induced cytotoxicity. This observation supports a model in which the ATF3–VASP axis contributes to enhanced platelet activation and subsequent neuronal damage. Of note, emerging evidence showed that VASP is closely related to neurological damage after ICH [57,58]. The above results fully demonstrate that the ATF3-VASP axis plays an important role in secondary brain injury after cerebral hemorrhage.

Vasodilator-stimulated phosphoprotein (VASP) is a key actin regulatory protein expressed in platelets, endothelial cells, and neurons. It is best known for its role in modulating cytoskeletal dynamics by serving as a substrate for cyclic nucleotide-dependent kinases (PKA and PKG), which phosphorylate VASP at key serine residues [59]. In platelets, the phosphorylation state of VASP is a critical determinant of its function: under basal conditions, non-phosphorylated VASP promotes actin polymerization and cytoskeletal reorganization, thereby facilitating platelet adhesion and aggregation. Following ICH, the extravasation of blood components into brain parenchyma triggers a cascade of inflammatory and thrombotic events. Activated platelets are central to this process as they adhere to damaged vascular endothelium and form microthrombi, which can obstruct microcirculation and exacerbate ischemia [60]. Moreover, platelet activation promotes the release of pro-inflammatory mediators and cytokines that amplify local inflammatory responses, recruit leukocytes, and disrupt the blood–brain barrier (BBB) [47]. This inflammatory milieu not only aggravates the initial insult but also contributes to secondary brain injury by promoting edema, oxidative stress, and neuronal apoptosis [48,49]. In the context of our findings, the upregulation of VASP by ATF3 likely enhances these detrimental platelet responses, thereby facilitating a vicious cycle where platelet activation further compromises microvascular integrity and neuronal survival.

Despite these compelling findings, this study presents several important limitations that should be acknowledged to contextualize its findings. Firstly, the analysis is based on the GSE24265 dataset, which is both small in size and over a decade old, potentially limiting statistical robustness and the generalizability of the results. Mechanistic conclusions regarding VASP-mediated platelet activation and neuronal damage are largely drawn from prior literature rather than direct experimental validation within the current work. Additionally, the autologous blood injection model used for inducing ICH in mice, while standard, does not fully replicate the pathophysiological complexity of spontaneous human ICH, lacking underlying vasculopathy. In vitro experiments further simplify the ICH environment by relying solely on heme exposure to model neuronal toxicity, omitting other critical blood components and inflammatory mediators involved in secondary brain injury. Finally, while the study suggests the ATF3-VASP pathway as a potential therapeutic target, it does not sufficiently address the translational challenges such as off-target effects or the difficulty of delivering therapeutics across the blood-brain barrier. Addressing these limitations would strengthen the scientific and translational impact of the study.

## Conclusion

In conclusion, our study establishes that increased ATF3 expression is a key mediator of secondary brain damage following ICH via the direct upregulation of VASP and subsequent platelet activation. These findings not only broaden our understanding of the molecular underpinnings of secondary neuronal injury but also identify the ATF3–VASP axis as a promising therapeutic target for mitigating post-hemorrhagic brain damage.

## Supporting information

S1 Table List of differentially expressed genes. (CSV)

S2 Table The result of GO enrichment. (CSV)

S3 Table The results of KEGG enrichment. (CSV)

S1 Fig Protein-protein interaction networks. (TIF)

S2 Fig The results of GO enrichment analysis. (TIF)

S1 Data Raw data. (DOCX)
