# Supplementary figures and images for: Integrated bioinformatics and experimental validation identify ATF3 as a key gene in secondary brain damage after intracerebral hemorrhage
Source: PLoS One. 2025 Jul 18;20(7):e0328530. doi: 10.1371/journal.pone.0328530 (PMC12273966; doi:10.1371/journal.pone.0328530)

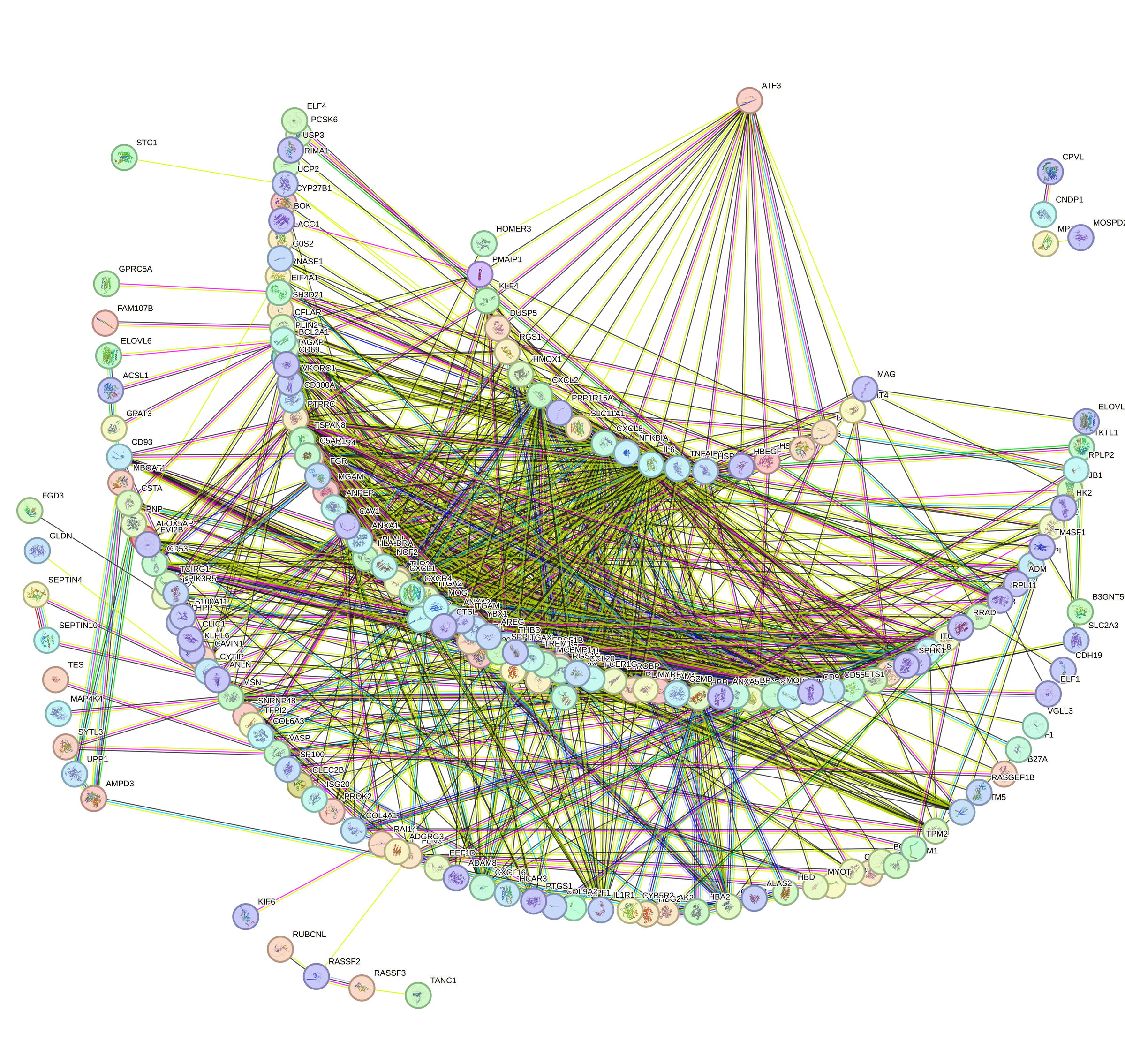

Supplement: S1 Fig — (TIF) [file pone.0328530.s004.tif]

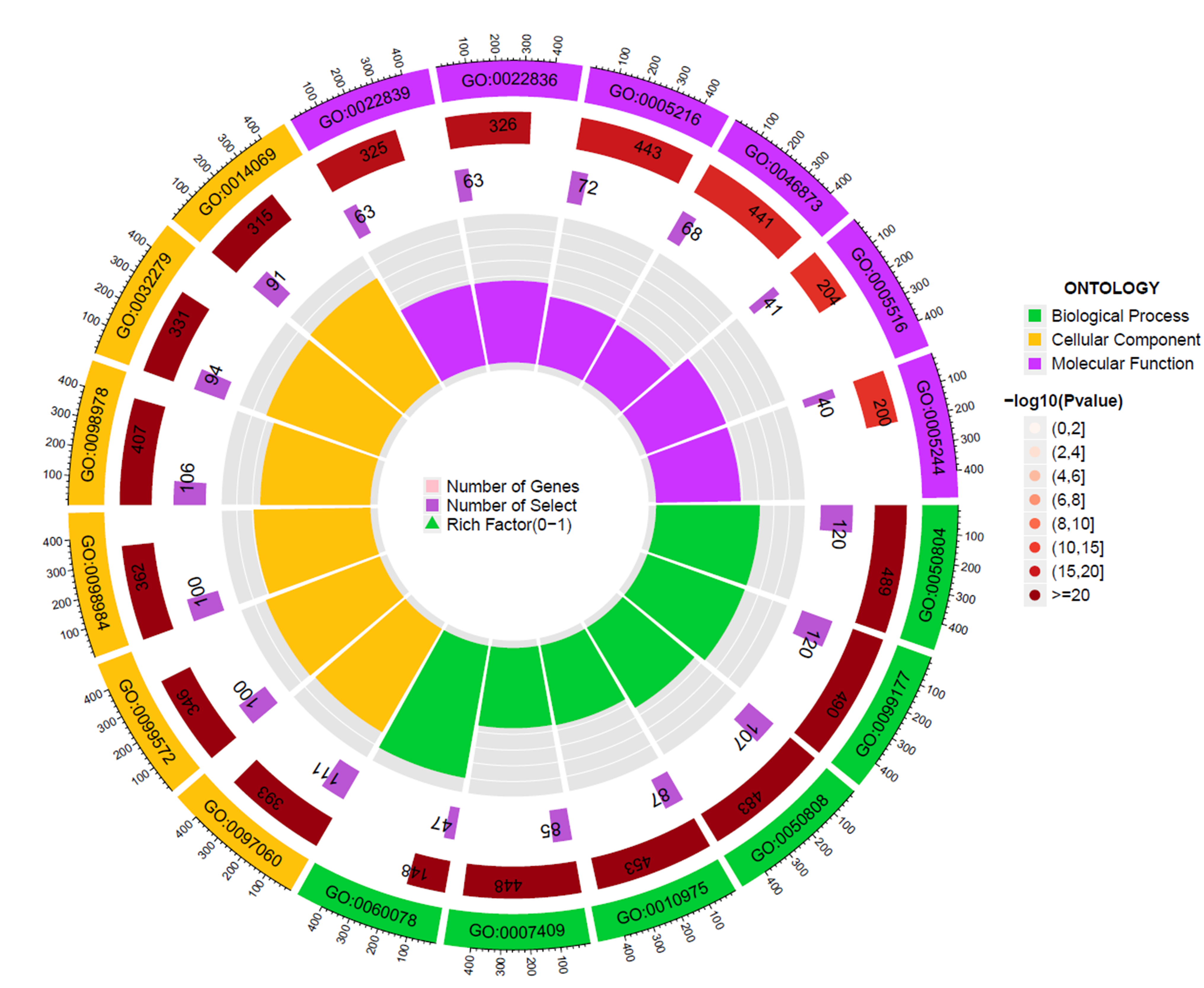

Supplement: S2 Fig — (TIF) [file pone.0328530.s005.tif]
