# Supplementary material for: Integrated bioinformatics and experimental validation identify ATF3 as a key gene in secondary brain damage after intracerebral hemorrhage
Source: PLoS One. 2025 Jul 18;20(7):e0328530. doi: 10.1371/journal.pone.0328530 (PMC12273966; doi:10.1371/journal.pone.0328530)
Supplement: S1 Data — (DOCX) [file pone.0328530.s006.docx]

Row data source

The raw data of Fig 1. comes from GEO

The raw data of Fig 2. comes from GEO

The raw data of Fig 3. comes from GEO

The raw data of Fig 4. comes from GEO

The raw data of Fig 5. comes from GEO

The raw data in figure 6 are the following:

Figure 6

Fig 6C.

| **group** | **sample (n)** | **ATF3/GAPDH (Mean±SD)** | **p** |
| --- | --- | --- | --- |
| Control | 3 | 0.485 ± 0.032 | 0.0015 |
| ICH | 3 | 1.000 ± 0.057 |  |

Fig 6E

| **group** | **heme (mM)** | **sample (n)** | **ATF3/GAPDH (Mean±SD)** | **p** |
| --- | --- | --- | --- | --- |
| Control | 0 | 3 | 4.015 ± 0.032 | 0.001 |
| Heme | 80 | 3 | 1.010 ± 0.057 |  |

Figure 7

Fig 7C

| **group** | **sample (n)** | **ATF3/Actin (Mean±SD)** | **pvalue** | **VASP/Actin (Mean±SD)** | **pvalue** |
| --- | --- | --- | --- | --- | --- |
| Control | 3 | 0.957 ± 0.008 | - | 1.158 ± 0.003 | - |
| ATF3 Overexpressed | 3 | 1.540 ± 0.046 | <0.001 | 3.397 ± 0.036 | <0.001 |

Fig 7F

| **group** | **sample (n)** | **ATF3/Actin (Mean±SD)** | **pvalue** | **VASP/Actin (Mean±SD)** | **pvalue** |
| --- | --- | --- | --- | --- | --- |
| Control | 3 | 0.977 ± 0.008 | - | 0.858 ± 0.003 | - |
| ATF3 Knockdown | 3 | 0.430 ± 0.015 | <0.01 | 0.211 ± 0.027 | <0.001 |

Fig 7I

| **Group** | **sample (n)** | **Relative luciferase activity (Mean±SD)** |
| --- | --- | --- |
| Control | 5 | 1.050 ± 0.054 |
| ATF3 Overexpressed | 5 | 4.055 ± 0.160 |
| ATF3 Knockdown | 5 | 0.389 ± 0.028 |

| **comparison** | **mean** | **pvalue** |
| --- | --- | --- |
| Control vs ATF3_OE | -3.005 | <0.0001 |
| Control vs ATF3_KD | 0.661 | <0.0001 |
| ATF3_OE vs ATF3_KD | 3.666 | <0.0001 |

Fig8 B

| **group** | **mean ± sd** |
| --- | --- |
| Control | 2.60 ± 0.42 |
| Heme | 10.59 ± 1.14 |
| ATF3 overexpression+Heme | 23.42 ± 4.18 |

| **comparison** | **t value** | **df** | **p value** |
| --- | --- | --- | --- |
| Control vs Heme | -14.69 | 5.08 | < 0.001 |
| Control vs ATF3 overexpression+Heme | -11.08 | 4.08 | < 0.001 |
| Heme vs ATF3 overexpression+Heme | -6.62 | 4.59 | < 0.001 |

Figure 9.

Fig 9A.

| Control | Vehicle | Blood | ATF3 ovexpression+Blood |
| --- | --- | --- | --- |
| -0.09 | 0.16 | 0.45 | 0.98 |
| -0.07 | 0.16 | 0.45 | 0.95 |
| -0.01 | 0.11 | 0.58 | 0.88 |
| -0.01 | 0.21 | 0.62 | 0.91 |
| -0.05 | 0.22 | 0.56 | 0.98 |

| **group** | **mean ± sd** |
| --- | --- |
| Control | -0.05 ± 0.04 |
| Vehicle | 0.17 ± 0.04 |
| Blood | 0.53 ± 0.08 |
| ATF3 overexpression+Blood | 0.94 ± 0.04 |

| **comparison** | **t value** | **df** | **p value** |
| --- | --- | --- | --- |
| Control vs Vehicle | -8.549 | 7.655 | < 0.001 |
| Control vs Blood | -11.560 | 5.824 | < 0.001 |
| Control vs ATF3+Blood | -24.110 | 7.749 | < 0.001 |
| Vehicle vs Blood | -8.980 | 6.683 | < 0.001 |
| Vehicle vs ATF3+Blood | -27.429 | 7.998 | < 0.001 |
| Blood vs ATF3+Blood | -10.170 | 6.554 | < 0.001 |

Fig9 B.

| Control | Vehicle | Blood | ATF3 ovexpression+Blood |
| --- | --- | --- | --- |
| 3 | 4 | 7 | 13 |
| 2 | 3 | 6 | 14 |
| 1 | 5 | 8 | 12 |
| 2 | 3 | 5 | 10 |
| 2 | 2 | 8 | 11 |

| **group** | **mean ± sd** |
| --- | --- |
| Control | 2.0 ± 0.7 |
| Vehicle | 3.4 ± 1.1 |
| Blood | 6.8 ± 1.3 |
| ATF3 overexpression+Blood | 12.0 ± 1.6 |

| **comparison** | **t value** | **df** | **p value** |
| --- | --- | --- | --- |
| Control vs Vehicle | -2.333 | 6.68 | 0.057 |
| Control vs Blood | -7.238 | 6.26 | < 0.001 |
| Control vs ATF3+Blood | -12.649 | 6.00 | < 0.001 |
| Vehicle vs Blood | -4.905 | 7.53 | 0.0012 |
| Vehicle vs ATF3+Blood | -10.316 | 7.80 | < 0.001 |
| Blood vs ATF3+Blood | -5.412 | 7.94 | 0.0006 |

Fig 9C

| Control | Vehicle | Blood | ATF3 ovexpression+Blood |
| --- | --- | --- | --- |
| 6 | 10 | 36 | 55 |
| 4 | 9 | 28 | 48 |
| 7 | 12 | 27 | 46 |
| 4 | 11 | 34 | 54 |
| 5 | 8 | 33 | 56 |

| **group** | **mean ± sd** |
| --- | --- |
| Control | 5.2 ± 1.3 |
| Vehicle | 8.0 ± 1.6 |
| Blood | 31.6 ± 3.9 |
| ATF3 overexpression+Blood | 51.8 ± 4.5 |

| **comparison** | **t value** | **df** | **p value** |
| --- | --- | --- | --- |
| Control vs Vehicle | -5.21 | 7.68 | 0.051 |
| Control vs Blood | -15.30 | 4.98 | < 0.001 |
| Control vs ATF3+Blood | -27.52 | 4.99 | < 0.001 |
| Vehicle vs Blood | -12.50 | 4.16 | < 0.001 |
| Vehicle vs ATF3+Blood | -21.47 | 4.99 | < 0.001 |
| Blood vs ATF3+Blood | -9.19 | 7.99 | < 0.001 |

Fig 9D

| Control | Vehicle | Blood | ATF3 downregulation+Blood |
| --- | --- | --- | --- |
| -0.09 | 0.16 | 0.65 | 0.28 |
| -0.07 | 0.26 | 0.55 | 0.25 |
| -0.01 | 0.18 | 0.68 | 0.28 |
| -0.01 | 0.11 | 0.52 | 0.21 |
| -0.05 | 0.2 | 0.56 | 0.29 |

| **group** | **mean ±sd** |
| --- | --- |
| Control | -0.05 ± 0.04 |
| Vehicle | 0.18 ± 0.06 |
| Blood | 0.59 ± 0.07 |
| ATF3 downregulation+Blood | 0.26 ± 0.03 |

| **comparison** | **t value** | **df** | **p value** |
| --- | --- | --- | --- |
| Control vs Vehicle | -7.31 | 6.48 | < 0.001 |
| Control vs Blood | -15.75 | 5.31 | < 0.001 |
| Control vs ATF3 down+Blood | -11.26 | 6.15 | < 0.001 |
| Vehicle vs Blood | -10.84 | 6.08 | < 0.001 |
| Vehicle vs ATF3 down+Blood | -3.80 | 6.60 | 0.0076 |
| Blood vs ATF3 down+Blood | 9.73 | 5.93 | < 0.001 |

Fig 9E

| Control | Vehicle | Blood | ATF3 downregulation+Blood |
| --- | --- | --- | --- |
| 4 | 6 | 9 | 4 |
| 1 | 3 | 6 | 8 |
| 1 | 4 | 8 | 5 |
| 3 | 3 | 9 | 5 |
| 3 | 2 | 8 | 6 |

| **group** | **mean ± sd** |
| --- | --- |
| Control | 2.4 ± 1.3 |
| Vehicle | 3.6 ± 1.5 |
| Blood | 8.0 ± 1.2 |
| ATF3 downregulation+Blood | 5.6 ± 1.5 |

| **comparison** | **t value** | **df** | **p value** |
| --- | --- | --- | --- |
| Control vs Vehicle | -1.325 | 7.88 | 0.222 |
| Control vs Blood | -7.746 | 6.77 | < 0.001 |
| Control vs ATF3 down+Blood | -3.162 | 7.88 | 0.014 |
| Vehicle vs Blood | -5.477 | 6.15 | 0.001 |
| Vehicle vs ATF3 down+Blood | -2.190 | 7.88 | 0.061 |
| Blood vs ATF3 down+Blood | 2.752 | 7.66 | 0.026 |

Fig 9F.

| Control | Vehicle | Blood | ATF3 downregulation+Blood |
| --- | --- | --- | --- |
| 5 | 9 | 36 | 25 |
| 4 | 7 | 38 | 18 |
| 8 | 12 | 27 | 26 |
| 6 | 13 | 34 | 24 |
| 4 | 8 | 32 | 18 |

| **group** | **mean ± sd** |
| --- | --- |
| Control | 5.4 ± 1.7 |
| Vehicle | 9.8 ± 2.6 |
| Blood | 33.4 ± 4.2 |
| ATF3 downregulation+Blood | 22.2 ± 3.9 |

| **comparison** | **t value** | **df** | **p value** |
| --- | --- | --- | --- |
| Control vs Vehicle | -3.19 | 6.84 | 0.016 |
| Control vs Blood | -14.29 | 4.93 | < 0.001 |
| Control vs ATF3 down+Blood | -8.15 | 5.75 | < 0.001 |
| Vehicle vs Blood | -11.14 | 4.88 | < 0.001 |
| Vehicle vs ATF3 down+Blood | -6.95 | 7.88 | < 0.001 |
| Blood vs ATF3 down+Blood | 4.87 | 7.99 | 0.001 |

Fig 9G

| Control | Vehicle | Blood | Overexpressed ATF3+Blood |
| --- | --- | --- | --- |
| 53 | 64 | 76 | 84 |
| 63 | 58 | 68 | 82 |
| 52 | 62 | 72 | 82 |
| 64 | 56 | 70 | 78 |
| 54 | 59 | 66 | 81 |

| **group** | **mean ± sd** |
| --- | --- |
| Control | 57.2 ± 5.8 |
| Vehicle | 59.8 ± 3.5 |
| Blood | 70.4 ± 4.3 |
| Overexpressed ATF3+Blood | 81.4 ± 2.3 |

| **comparison** | **t value** | **df** | **p value** |
| --- | --- | --- | --- |
| Control vs Vehicle | -0.85 | 6.01 | 0.428 |
| Control vs Blood | -4.44 | 6.96 | 0.003 |
| Control vs ATF3+Blood | -8.90 | 4.87 | < 0.001 |
| Vehicle vs Blood | -4.17 | 6.56 | 0.004 |
| Vehicle vs ATF3+Blood | -11.45 | 4.73 | < 0.001 |
| Blood vs ATF3+Blood | -5.70 | 5.96 | 0.001 |

Fig 9H

| Control | Vehicle | Blood | ATF3 downregulation+Blood |
| --- | --- | --- | --- |
| 52 | 54 | 76 | 64 |
| 54 | 58 | 78 | 62 |
| 52 | 62 | 72 | 62 |
| 58 | 56 | 70 | 58 |
| 54 | 58 | 66 | 61 |

| **group** | **mean ± sd** |
| --- | --- |
| Control | 54.0 ± 2.4 |
| Vehicle | 57.6 ± 3.0 |
| Blood | 72.4 ± 4.8 |
| ATF3 downregulation+Blood | 61.4 ± 2.2 |

| **comparison** | **t value** | **df** | **p value** |
| --- | --- | --- | --- |
| Control vs Vehicle | -2.10 | 7.63 | 0.070 |
| Control vs Blood | -7.67 | 5.88 | < 0.001 |
| Control vs ATF3 down+Blood | -5.08 | 7.94 | 0.001 |
| Vehicle vs Blood | -5.41 | 6.06 | 0.001 |
| Vehicle vs ATF3 down+Blood | -2.33 | 6.42 | 0.056 |
| Blood vs ATF3 down+Blood | 5.26 | 6.58 | 0.001 |

Fig 9I

| Vehicle+Blood | ATF3 overexpressed+Blood | ATF3 downregulation+Blood |
| --- | --- | --- |
| 6 | 6 | 8 |
| 8 | 6 | 6 |
| 5 | 5 | 8 |
| 8 | 5 | 8 |
| 6 | 6 | 9 |
|  |  |  |

| **group** | **mean ± sd** |
| --- | --- |
| Vehicle+Blood | 6.6 ± 1.1 |
| ATF3 overexpressed+Blood | 5.6 ± 0.5 |
| ATF3 downregulation+Blood | 7.8 ± 1.1 |

| **comparison** | **t value** | **df** | **p value** |
| --- | --- | --- | --- |
| Vehicle+Blood vs ATF3 overexpressed+Blood | 1.96 | 6.06 | 0.096 |
| Vehicle+Blood vs ATF3 downregulation+Blood | -1.70 | 7.99 | 0.129 |
| ATF3 overexpressed+Blood vs ATF3 downregulation+Blood | -4.24 | 5.54 | 0.006 |
